# Chronic anemia due to watermelon stomach

**DOI:** 10.4103/0256-4947.60524

**Published:** 2010

**Authors:** Baris Yildiz, Cenk Sokmensuer, Volkan Kaynaroglu

**Affiliations:** a From the Department of General Surgery, Hacettepe University, Ankara, Turkey; b From the Department of Pathology, Hacettepe University, Ankara, Turkey

## Abstract

Antral gastric vascular ectasia is a rare cause of chronic anemia. When encountered, the diagnosis is usually delayed. Endoscopic findings are well established, although radiologic findings are not. Patients respond well to surgery. Our case was of a 62-year-old female with chronic anemia who required multiple blood transfusions and iron replacement therapy, without significant response. Computed tomography revealed a focal thickening of the gastric antrum. Endoscopy showed vascular ectasia between the antrum and corpus. The patient underwent gastrectomy. We reviewed the literature on gastric angiodysplasia and have presented our unique tomography findings in this first report on a novel association between ectopic pancreas and gastric angiodysplasia.

Although the differential diagnosis of chronic anemia might seem straightforward, intriguing choices can be encountered during its evaluation. Finding the etiology of anemia can be challenging, especially in females and elderly patients. Evaluation starts with a basic physical examination, but may even require surgical exploration in difficult cases. The insidious nature of the chronic blood loss renders such patients symptomless for prolonged periods despite very low hemoglobin values. Rare disease associations and unusual clinical findings should be kept in mind for cases with obscure etiologies. We describe a rare cause of chronic anemia with a unique association.

## CASE

A 62-year-old female patient presented to our outpatient clinic with fatigue. She had been followed for anemia, with changing hemoglobin values, between 7 and 11 g/dL (normal reference range, 12-18 g/dL), for over six years in different centers. An occult fecal blood test was positive and fecal parasite analysis was negative. She had received multiple tranfusions in the previous six years and had been started on oral iron replacement therapy before presenting to our clinic. Colonoscopy revealed normal findings. Two years previously she was diagnosed with antral vascular ectasia by endoscopy. Endoscopic cauterization of the bleeding foci had been performed twice.

**Figure 1:**
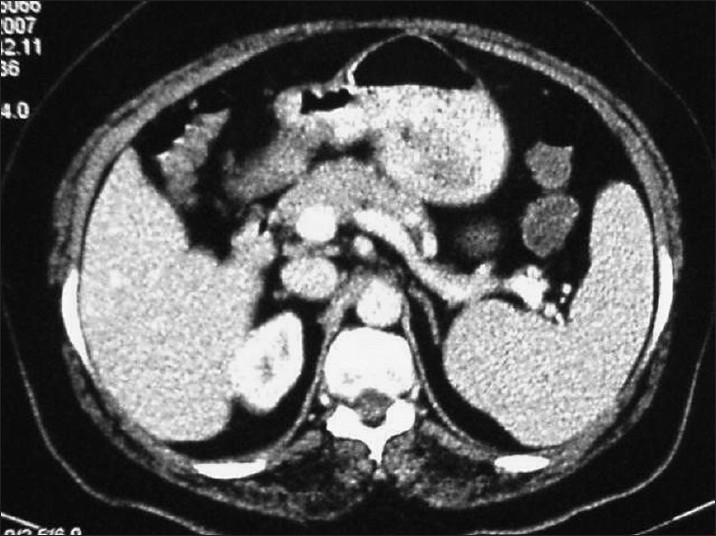
CT scan showing focal thickening of the gastric antrum.

At the latest presentation, a CT scan showed focal thickening of the gastric antrum (Figure 1). A repeat endoscopy revealed vascular ectasia between the antrum and corpus. Her hemoglobin level was 8.8 g/dL. The only serum biochemistry abnormality was a high indirect bilirubin of 1.55 (normal reference range, 0.10-1.20 mg/dL). Her physical examination was normal except for laparoscopic cholecystectomy incision scars.

**Figure 2:**
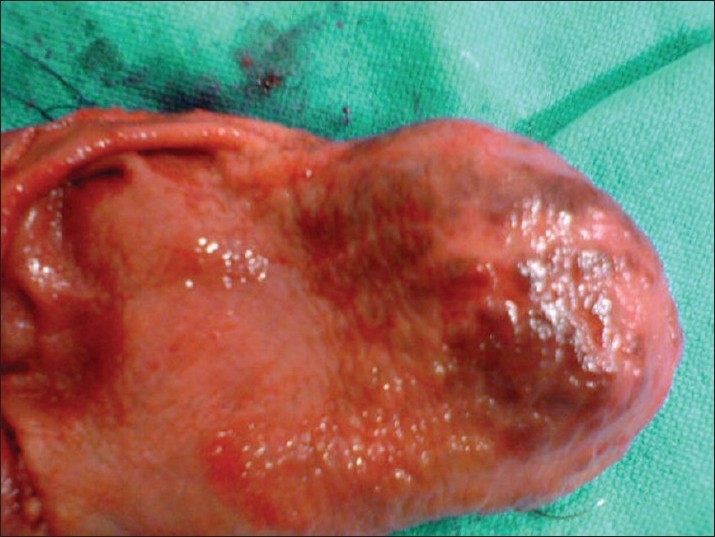
Columns of angiodysplasia.

**Figure 3:**
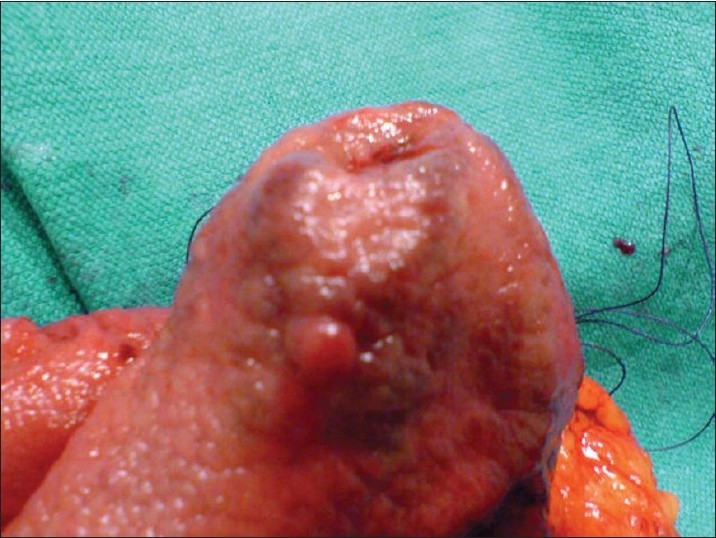
Close-up view of the angiodysplasia columns with antrum everted.

Considering the intractable course of her disease, she underwent surgery. During the operation, the abdominal exploration was normal. After gastrotomy, columns of angiodysplasia were seen running from the antrum to the corpus (Figures 2 and 3). Hemigastrectomy and a Billroth 2 procedure were performed. The postoperative course of the patient was uneventful. She was discharged with normal oral intake.

**Figure 4:**
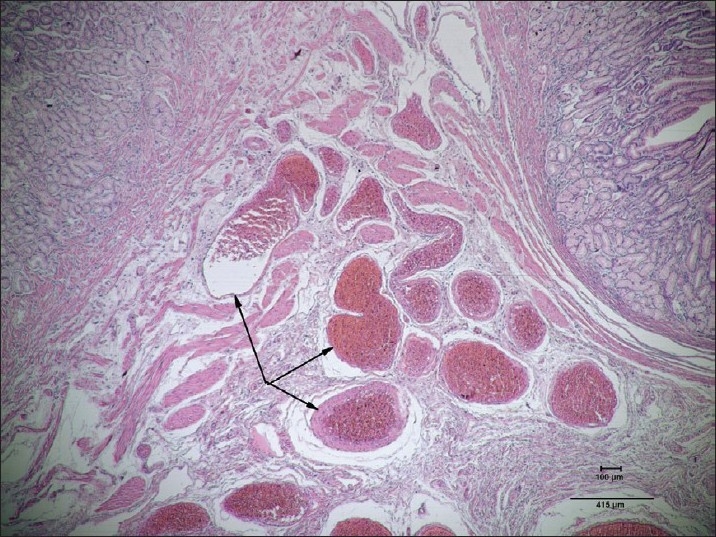
Submucosal abnormal vascular dilatation and structures that have penetrated into the mucosa, (hematoxylin and eosin, ×40).

**Figure 5:**
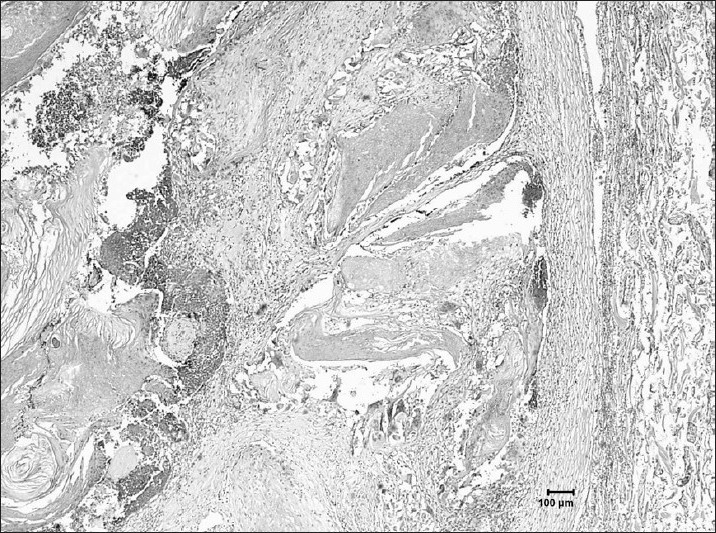
The lesion is well demarcated from the surrounding fibroconnective tissue, (hematoxylin and eosin, ×40).

The pathology report showed mucosal and submucosal malformation of the dilated vascular structures, which were compatible with angiodysplasia (Figures 4 and 5). A focus on ectopic pancreas was seen on the antrum.

## DISCUSSION

Common causes of upper gastrointestinal hemorrhage include peptic ulcer disease, esophageal varices, gastritis, esophagitis, and Mallory-Weiss tears. One of the unusual causes is gastric antral angiodysplasia which results in major chronic gastrointestinal hemorrhage. Apart from anemia, gastric outlet obstruction is also reported in these patients.^1^ The pathognomonic histological features include foveolar hyperplasia, vascular ectasia with clots, and fibromuscular spindle cell hyperplasia of the lamina propria.^2^,^3^ Chronic unexplained anemia resulting from bleeding of abnormal gastric vessels is a well recognized phenomenon but the condition is poorly documented and its etiology and pathogenesis are unclear.^4^–^6^

This syndrome was first recognized endoscopically by Wheeler et al. in 1979, but it was Jabbari et al. who first described three patients of their own and identified four cases from published reports.^5^,^7^ The etiology of this condition remains unknown. These lesions can be seen in cirrhosis, in up to 30% of the patients, in two forms, as portal hypertensive gastropathy and gastric antral vascular ectasia, which are two seperate entities based on endoscopic and histolpathological findings.^8^ In this setting, bleeding is usually slow and insidious and rarely massive and life threatening.^9^–^11^

In the absence of cirrhosis, gastric antral vascular ectasia is a distinct clinicopathological entity. The most widely accepted theory for the etiology focuses on the recurrent episodes of antral mucosal prolapse or an intussusception into the pylorus that would lead to chronic trauma and to consequent fibromuscular hyperplasia of the antral wall. Vasoactive substances as well as increased gastrin levels may play an important role in the pathophysiology, promoting vasodilatation and antral motility.^12^ The resemblance of the histological findings to those of prolapse of the rectal mucosa, and those seen at stomal sites, support this theory.^3^

In the diagnosis, endoscopic findings are crucial and columns of dilated ectatic vessels on the summits of prominent antral rugal folds are characteristic. These appearances, however, can easily be misinterpreted as moderate-to-severe gastritis.^13^

Although recommended by most authorities, radiological investigations, including barium meal and mesenteric angiography, often fail to make the diagnosis, and identification of the cause of blood loss is usually delayed for many years.^14^,^15^ These patients characteristically respond well to antrectomy and Bilroth anastomosis. Long-term corticosteroids and heat probe therapy have been reported as a safe options in older, medically unfit patients, while others have advocated endoscopic laser treatment.^16^,^17^ Our case shows once again that in the workup for differential diagnosis of chronic anemia, gastric angiodysplasia should be investigated. Although there are not many reports on the characteristic CT findings in this entity, our case supports the CT findings of other authors. We feel that this should prompt a planned radiological research to identify characteristic appearances on CT, which could be used for patients not suitable for endoscopy. One other interesting finding was ectopic pancreas, in our pathology specimen. There are no other reports in the literature on this association.
